# Adventitial Cyst of the Popliteal Artery

**DOI:** 10.5334/jbsr.2109

**Published:** 2020-07-03

**Authors:** Margot Stagnetto, Patrick Mailleux

**Affiliations:** 1 Clinique St Luc Bouge, BE

**Keywords:** adventitial cyst, popliteal artery, claudication, Doppler

## Abstract

**Teaching Point:** This clinical case shows the importance of also examining the arteries when investigating suspected deep venous thrombosis.

## Case Report

**Figure 1:**
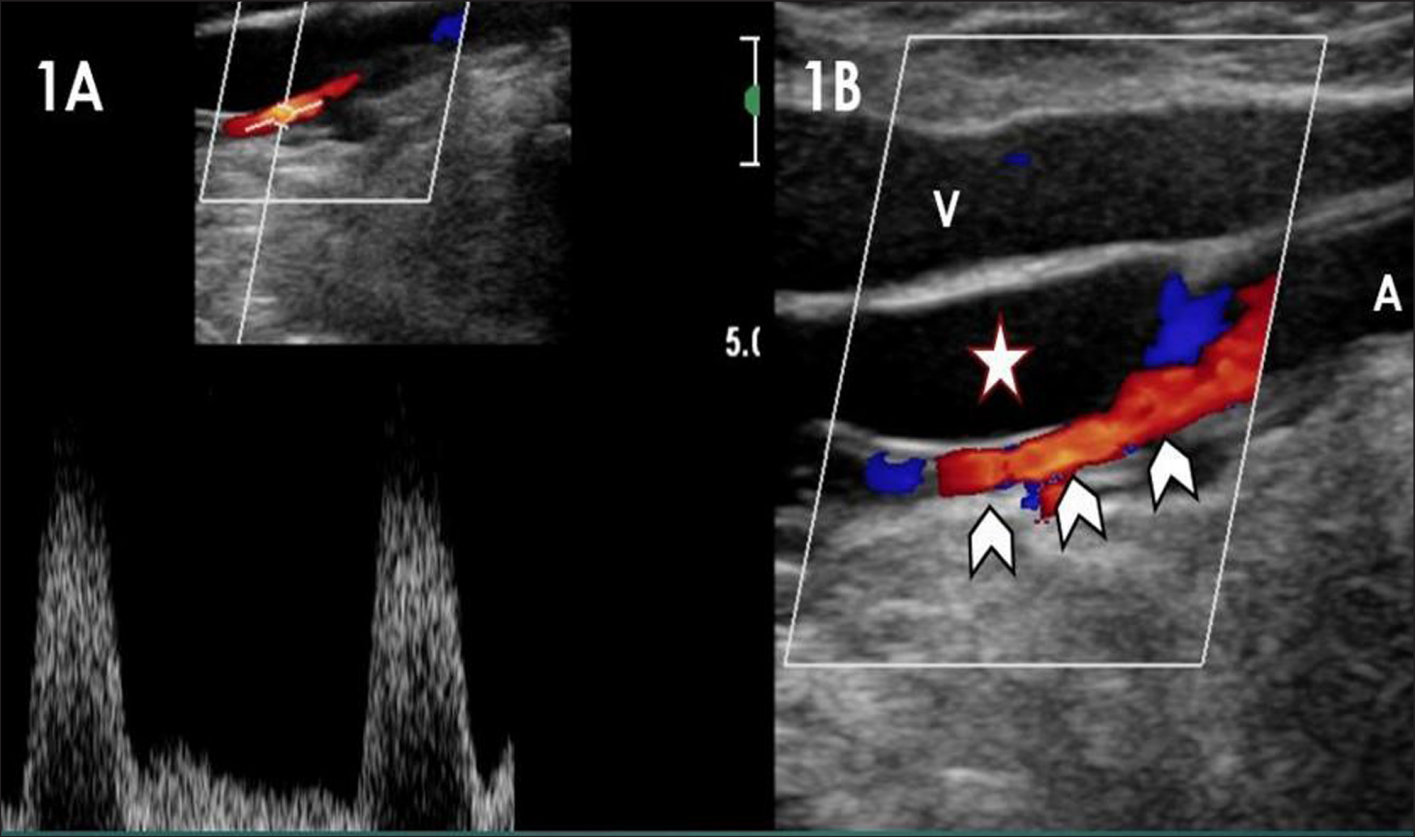


**Figure 2:**
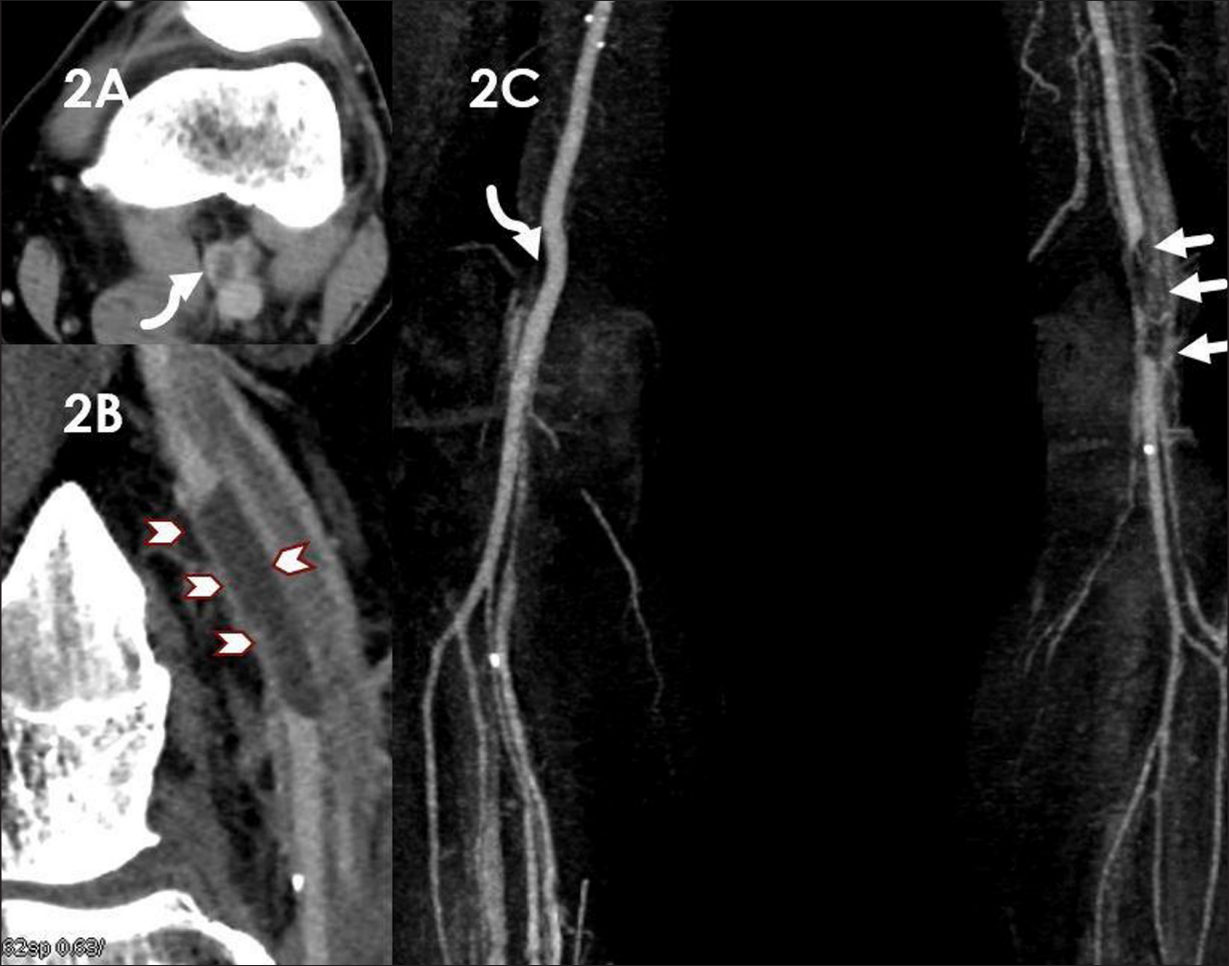


A 54-year-old patient, very active and a smoker, was sent for ultrasound of the knee and calf to exclude deep venous thrombosis (DVT). Upon specific questioning, the patient revealed that for four days he had been suffering from constant posterior knee pain while calf pain actually occurred only during walking. The popliteal vein was depressible and DVT was excluded, as was rupture of a popliteal cyst. Incidentally on Doppler ultrasound, the popliteal artery showed flow acceleration up to 350 cm/sec, due to a tight stenosis (Figure 1A). It was caused by a sharply delineated intra-parietal anechoic lesion (star in Figure 1B). Contrast-enhanced computed tomography (CT) showed a 4 cm-length adventitial cyst reducing the diameter of the popliteal artery (curved arrows in Figure 2A, arrowheads in Figure 2B). Figure 2C is a MIP coronal reconstruction of the popliteal arteries. A slight extrinsic deformation can be seen on the right popliteal artery (curved arrow), related to a synovial cyst with no arterial wall extension. The left popliteal artery shows the typical “scimitar” appearance represented by an asymmetric narrowing caused by the adventitial cyst (straight arrows). Treatment was a surgical by-pass with a venous graft, granting an uneventful recovery.

## Comment

Adventitial cyst is a rare vascular disease that causes peripheral artery stenosis or occlusion by luminal compression. It mainly affects men aged 40 to 50 years old, and the popliteal artery is the most frequently involved vessel (85%). The incidence is estimated at 1:1200 in the case of arterial stenosis around the knee. Usually it affects the popliteal artery unilaterally. The clinical presentation is often intermittent claudication in patients at low risk of vascular disease whose clinical diagnosis is therefore difficult [1]. On imaging, confirmation of clinical diagnosis by interventional arteriography was earlier considered as the standard of reference. Nevertheless, Doppler ultrasound has become, as in our case, a non-invasive diagnostic method sufficient for the diagnosis.

Contrast CT and magnetic resonance imaging are useful for assessing the detailed morphology of the cysts and the stenosis and can additionally reveal a communication between the cyst and an adjacent joint. On contrast CT, the cyst is commonly of “water density”, causing an “hourglass” or “scimitar” narrowing of the vessel lumen. The cysts appear respectively hypointense and hyperintense on T1- and T2-weighted magnetic resonance imaging.
